# Calcium and strontium isotopes in extant diapsid reptiles reflect dietary tendencies—a reference frame for diet reconstructions in the fossil record

**DOI:** 10.1098/rspb.2024.2002

**Published:** 2025-01-08

**Authors:** Michael Weber, Katrin Weber, Daniela E. Winkler, Thomas Tütken

**Affiliations:** ^1^ Institute for Geosciences, Johannes Gutenberg University, Mainz, Germany; ^2^ Zoological Institute, Kiel University, Kiel, Germany

**Keywords:** Lepidosaurs, crocodiles, trophic level, enamel, bone, dental wear

## Abstract

Dietary preferences of extant reptiles can be directly observed, whereas diet reconstruction of extinct species typically relies on morphological or dental features. More specific information about the ingested diet is contained in the chemistry of hard tissues. Stable isotopes of calcium and strontium show systematic fractionations between diet and skeletal bioapatite, which is applied for diet and trophic-level reconstructions of extant and extinct vertebrate species. Here, we present the first comprehensive analysis of stable calcium and strontium isotopes of bones and teeth from 28 extant reptiles, including lepidosaurs and archosaurs (crocodilians) with distinct herbivorous to faunivorous feeding behaviour, establishing a dietary reference frame. Both calcium and strontium isotopes exhibit systematic offsets between dietary groups, with insectivores having the highest, herbivores intermediate and carnivores the lowest calcium and strontium isotope values. Although the isotopic trophic-level effect is similar to mammals, the absolute calcium isotope values in reptiles are more positive in each diet category. Combining isotopic data with dental microwear texture analysis enables a refined understanding of reptile feeding ecology and the identification of durophagous diets. This toolbox opens new possibilities for improved dietary reconstructions of extinct taxa, such as dinosaurs and other non-mammalian species in the fossil record.

## 1. Introduction

All extant reptiles belong to the clade Amniota, which comprises most terrestrial tetrapod vertebrates today. The earliest evidence of amniotes in the fossil record dates back to the Lower Pennsylvanian (about 315 million years ago (Mya)) with the earliest known reptile, *Hylonomus lyelli* [1]. Reptiles can be further classified into Anapsida, Euryapsida and Diapsida, with the latter group divided into the two monophyletic clades of lepidosaurs and archosaurs, including lizards, crocodiles and birds.

When referring to ‘reptiles’ in colloquial language, usually the extant lepidosaurs, crocodylians and turtles are included. Without delving into the current phylogenetic discussion about whether or not reptiles are a monophylum, or more likely a paraphyletic grade or even wholly polyphyletic grouping [2–5], for this article we use the term ‘reptile’ when referring to extant lepidosaurs and crocodylians, and are excluding extant birds and turtles for practical reasons, as they both do not have teeth.

The oldest known ancestors of diapsid reptiles lived about 302 Mya in the Upper Pennsylvanian and were described as *Petrolacosaurus* [6]. Early reptiles shared common morphological features in their skulls, jaws and teeth, indicating a tendency towards insectivorous feeding traits [7]. However, the precise timing of the dietary shift from insectivory to carnivory and herbivory in reptiles remains unclear. The earliest herbivorous tetrapods, known from the latest Carboniferous and early Permian [8,9], were synapsids and thus not direct ancestors of extant reptiles.

Today, diapsids, including birds, show a wide range of dietary preferences [10–13] ranging from herbivorous to faunivorous feeding habits, but also comprising dietary specializations. For instance, they include specialized herbivores, such as the algaevorous *Amblyrhynchus cristatus*, insectivorous chameleons and swallows, ovivorous snakes and Gila monsters (*Heloderma* spp.), various omnivorous taxa and carnivorous apex predators like *Crocodylus porosus*, *Varanus komodoensis* and *Falconiformes*. These dietary differences are reflected in distinct adaptations in dental (excluding birds) and skeletal morphology, even among species with similar dietary habits. This diversity in feeding ecology complicates the reconstruction of dietary preferences in the fossil record, as behavioural observations are not possible and findings of fossil gut contents or bite marks are rare [8,14].

Besides morphological and biomechanical studies, chemical and dental wear proxy data can help to reconstruct the diet of extinct species and characterize the material and chemical (i.e. isotopic) properties of their ingesta. However, to fully understand and interpret such proxy data from fossil specimens, it is essential to study modern, closely related species with known feeding habits to establish a dietary reference framework for comparison and accurate diet assignment.

Dental microwear texture analysis (DMTA) is a non-destructive, mechanical dietary proxy that has previously been used to reconstruct dietary preferences and feeding habits in various extant and extinct vertebrate species. Although this technique was originally developed for and primarily applied to mammalian species [15,16], due to their pronounced diet–tooth interaction during chewing, recent studies have successfully also applied DMTA to non-mammalian species, including fish and reptiles, and even extinct species such as pterosaurs and dinosaurs [10,17–21]. Although diet-related dental microwear can be preserved in the fossil record, identifying suitable enamel surface areas can be challenging due to post-mortem taphonomic alterations or modifications by preparation and conservational efforts [22,23].

Besides this mechanical dietary proxy, chemical proxies such as trace element and isotope compositions of skeletal remains can be used to reconstruct the diet of extinct taxa. The isotope composition of ingested food and drinking water determines the isotope composition of the body fluids and, ultimately, the isotope composition of the skeletal and dental hard tissues (i.e. bones and teeth), which can thus be used to reconstruct the diet and trophic ecology of vertebrates [24–26]. Bones are formed during skeletal growth in early ontogeny and are constantly remodelled throughout an individual’s lifetime by dissolution and reprecipitation processes [27]. In contrast, most modern terrestrial vertebrates form and mineralize their teeth within a relatively short time span, ranging from months up to several years early in an animal’s life, though depending on species and tooth position, and dental tissues are not remodelled afterwards [28].

Most modern lepidosaurs have a pleurodont dentition and continuously replace their teeth, known as polyphyodonty [29,30]. Similarly, the thecodont Archosauromorpha, such as crocodiles, constantly shed their teeth [31]. A noteworthy exception are lepidosaurs with an acrodont dentition, such as Chamaeleonidae and Agamidae, which do not replace their teeth during adulthood [31]. Therefore, unlike in diphyodont mammals, the chemical composition of reptilian teeth often does not reflect early ontogenetic stages.

In vertebrate palaeobiology and palaeoecology, a common approach to assess the diet of fossil taxa is the analysis of stable isotope compositions of their skeletal hard tissues such as dental tissues, especially of the taphonomically robust enamel. Stable isotope compositions are usually expressed as abundance ratios (e.g. ^13^C/^12^C or ^44^Ca/^42^Ca) relative to a reference material using the ‘delta’ notation (e.g. δ^13^C or δ^44/42^Ca). The abundance of stable isotopes of a given element in nature typically varies in the per mille range but can be systematically changed within organisms by various mass-dependent isotope fractionation effects during food processing, metabolism and biomineralization. Thus, the stable isotope composition can be used to reconstruct trophic-levels and diet categories of animals [25,32,33].

For more than four decades, classical light stable isotopes of carbon (δ^13^C) and nitrogen (δ^15^N) have been used to reconstruct diet and food webs [34,35] as well as to better understand dietary habits and niche partitioning in extant reptiles [36,37]. However, these light stable isotopes, which are primarily bound to the organic collagen phase of bone and dentin, are prone to post-mortem alteration (i.e. organic matter degradation) and diagenesis [38], limiting their use in reconstructing the trophic ecology of past ecosystems to Holocene‒Late Pleistocene settings with good collagen preservation [39,40]. Advances in analytical instrumentation in recent decades now allow the analysis of non-traditional metal stable isotopes, such as calcium (δ^44/42^Ca), zinc (δ^66^Zn) and strontium (δ^88/86^Sr) in enamel bioapatite, applicable to both modern and fossil ecosystems [25,32,41–44]. Calcium isotopes are a promising tool for diet reconstructions for fossil vertebrates. Due to the high calcium abundance in skeletal remains (~38 wt% calcium in the bioapatite of bone and enamel), this isotope system appears resistant to diagenetic alteration, especially in tooth enamel [45], and capable of preserving diet-related information over geological timescales [46–48].

The metabolism and incorporation of calcium during biomineralization processes into hard tissues results in strong biological isotope fractionation. The lighter ^42^Ca is preferentially incorporated into the bioapatite over ^44^Ca, leading to a decreasing ^44^Ca/^42^Ca (i.e. lower δ^44/42^Ca values) in the hard tissue compared with the ingesta. This causes a systematic decrease in δ^44/42^Ca along the food chain with increasing trophic-level by about −0.30‰ for terrestrial tetrapods [48–53].

Within the mineral lattice of hydroxylapatite, calcium can be substituted by a variety of trace elements with similar chemical properties, such as ion radius and charge [54]. Strontium is one such non-essential trace element which exhibits a similar isotope fractionation effect as calcium within an organism. With increasing trophic-level, the δ^88/86^Sr value systematically decreases, making stable strontium isotopes a useful complementary trophic-level proxy [41,44,55]. However, the application of δ^88/86^Sr for dietary reconstruction is limited to the aforementioned few studies and was only recently applied to a fossil food web [44]. In contrast, the radiogenic strontium isotope ratio of ^87^Sr/^86^Sr is a well-established proxy for provenance studies and has been used for about four decades [56]. This approach involves comparing the ^87^Sr/^86^Sr ratios of animal tissues (e.g. bone–tooth pairs) with the bioavailable ^87^Sr/^86^Sr signatures of the local geology and fauna. This comparison can differentiate between local and non-local foraging individuals and can thus be used to reconstruct provenance and migration during an individual’s life history [57,58] by analysing intra-individual ^87^Sr/^86^Sr changes in dental and skeletal tissues [59]. However, the ^87^Sr/^86^Sr ratio is not measurably fractionated between diet and (hard-) tissue, and thus cannot be used to reconstruct dietary preferences (i.e. trophic-level differences), unlike the stable strontium isotope value δ^88/86^Sr [41,43,55,60].

Although stable calcium and, to a lesser extent, stable strontium isotopes have been used for over a decade to reconstruct dietary traits, most research has focused on mammalian species [55,61], marine ecosystems [49,62], or archaeological and fossil food webs [41,47,51,52]. Currently, there is no systematic study addressing the application of both stable calcium and strontium isotope systems as a trophic-level proxy for reptiles. This study aims to assess stable calcium and strontium isotopes as combined trophic-level proxies for extant diapsid reptiles (Lepidosauria and Archosauria) and to evaluate their potential for dietary reconstruction. Additionally, we compare stable calcium and strontium isotope data with previously published DMTA from the same individuals [10] to combine these chemical and mechanical dietary proxies and explore their potential for refined diet reconstructions. Overall, we aim to address the following research questions:

1. Is there a systematic offset in δ^44/42^Ca or δ^88/86^Sr between bone and enamel of the same individual, and does the type of feeding category influence such an offset?
2. Can different faunivorous feeding categories (i.e. carnivory, insectivory, ovivory) and herbivores be differentiated using δ^44/42^Ca and/or δ^88/86^Sr?
3. Do reptiles and mammals of the same feeding category have similar δ^44/42^Ca and δ^88/86^Sr values, and are trophic-level differences thus comparable across taxa?
4. Does the combination of δ^44/42^Ca and δ^88/86^Sr, together with dental wear texture data from the same teeth, provide further details about dietary habits and enable refined reconstructions of the feeding ecology?

## 2. Material and methods

### (a) Tooth and bone samples

Overall, 78 bone and tooth specimens from 28 extant reptilian species were sampled from zoological collections of the Senckenberg Forschungsinstitut und Naturmuseum Frankfurt (Germany) (SMF), Zoologisches Forschungsmuseum Alexander Koenig Bonn (Germany) (ZFMK) and Staatliches Museum für Naturkunde Stuttgart (Germany) (SMNS) and analysed for their calcium and strontium isotope compositions in this study. In total, the dataset includes 28 taxa assigned to six different diet categories: (1) herbivores, (2) insectivores, (3) omnivores with mixed diets of different proportions of plant, invertebrate and vertebrate prey, (4) ovivores, (5) durophages and (6) carnivores, including Varanidae, as well as Crocodylia (electronic supplementary material, table S1).

### (b) Analysis of calcium and strontium isotopes

Sample processing follows the methods described in Weber *et al*. [46]. Between 0.3 and 10 mg of sampled material were dissolved using distilled concentrated nitric acid and purified for calcium and strontium using the *prep*FAST-MC (ESI Elemental Scientific, Omaha, NE, USA) system. Calcium and strontium isotope analyses were performed separately using a Neptune Plus multi-collector inductively coupled plasma mass spectrometer (Thermo Fisher Scientific, Bremen, Germany) coupled with an Apex Omega HF (ESI Elemental Scientific, Omaha, NE, USA) desolvator system at the Institute for Geosciences, Mainz, following the methods described in Tacail *et al*. [61] and Weber *et al*. [63]. Results are presented as delta values for calcium (δ^44/42^Ca, relative to NIST SRM (National Institute of Standards and Technology Standard Reference Material) 915a) and strontium (δ^88/86^Sr, relative to NIST SRM 987). In addition, the ^87^Sr/^86^Sr ratio was measured within the same run as δ^88/86^Sr and samples were normalized to a NIST SRM 987 ratio of 0.710248 [64]. Further details are provided in the electronic supplementary material.

## 3. Results

### (a) Differences between enamel and bone from the same individuals

**Figure 1.**
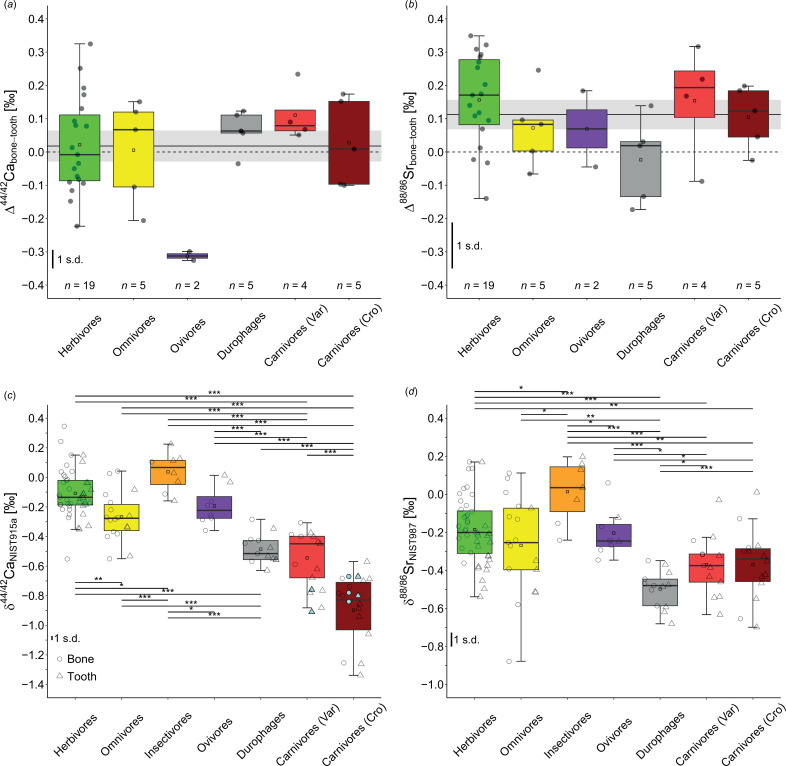
Difference between bone and tooth δ^44/42^Ca values of extant reptiles expressed as Δ^44/42^Ca_bone–tooth_ (*a*) and δ^88/86^Sr values expressed as Δ^88/86^Sr_bone–tooth_ (*b*). All bone–tooth pairs were obtained from the same individuals. Mean values for the bone–tooth offset are indicated as black lines with their respective uncertainties given as grey shading. A dashed black line represents no bone–tooth offset. The average Δ^44/42^Ca_bone–tooth_ yields 0.02 ± 0.05‰ (2 s.e., *n* = 40) and Δ^88/86^Sr_bone–tooth_ yields 0.11 ± 0.04‰ (2 s.e., *n* = 40). δ^44/42^Ca (*c*) and δ^88/86^Sr (*d*) values for bone (circles) and tooth (triangles) samples (electronic supplementary material, table S1) presented as boxplots for different diet categories. The carnivorous species have been divided into two groups (Varanidae and Crocodylia). Significance levels are defined as follows: *p < 0.05, **p < 0.01, ***p < 0.001. Mean propagated uncertainties from individual bone and tooth analyses for both isotope systems are provided at the 1 s.d. level. Literature δ^44/42^Ca data [48,53] is shown in light blue symbols for comparison.

δ^44/42^Ca and δ^88/86^Sr values and ^87^Sr/^86^Sr ratios were obtained from bone and enamel of 40 individuals from 15 different species, covering all feeding categories except for insectivores (electronic supplementary material, table S1). For both stable isotope systems, the bone–tooth offset shows a normal distribution (electronic supplementary material, table S4), ranging from −0.33 to +0.33‰ for calcium isotopes and from −0.18 to +0.35‰ for strontium isotopes. For calcium isotopes (figure 1*a*), the largest variation is observed in herbivorous reptiles, which include various Lepidosauria taxa. The average Δ^44/42^Ca_bone–tooth_ difference for herbivores is 0.02 ± 0.07‰ (2 s.e., *n* = 19). Omnivorous taxa show an average Δ^44/42^Ca_bone–tooth_ difference of 0.01 ± 0.12‰ (2 s.e., *n* = 5) and the durophagous taxa show 0.06 ± 0.05‰ (2 s.e., *n* = 5). Carnivorous taxa, divided into Varanidae (excluding the durophagous *V. niloticus*) and Crocodylia, show an average Δ^44/42^Ca_bone–teeth_ difference of 0.03 ± 0.11‰ (2 s.e., *n* = 5) for Crocodylia and 0.11 ± 0.07 (2 s.e., *n* = 4) for Varanidae. All groups are statistically indistinguishable from zero (i.e. no difference between bone and teeth pairs based on a one-sample *t*‐test, electronic supplementary material, table S4). In contrast, both ovivorous *Heloderma* specimens exhibit a significant offset of −0.31 ± 0.02‰ (2 s.e., *n* = 2, *p* < 0.05). The whole dataset shows no significant bone–tooth difference, with an average Δ^44/42^Ca_bone–tooth_ value of 0.02 ± 0.05‰ (2 s.e., *n* = 40) or 0.04 ± 0.04‰ excluding the *Heloderma* specimens (2 s.e., *n* = 38).

Stable strontium isotope data (δ^88/86^Sr) from the same individuals show a more pronounced difference between bone and teeth (figure 1*b*). Herbivores and varanids show the largest offset of 0.16 ± 0.06‰ (2 s.e., *n* = 19) and 0.15 ± 0.09‰ (2 s.e., *n* = 4), respectively, followed by the carnivorous Crocodylia with 0.10 ± 0.08‰. Omnivorous and ovivorous species show similar differences, with omnivores at 0.07 ± 0.09‰ (2 s.e., *n* = 5) and ovivores at 0.07 ± 0.16‰ (2 s.e., *n* = 2). Among herbivores, the marine algaevorous *A. cristatus* displays large positive offsets between bone and tooth (0.25 ± 0.07‰, 2 s.e., *n* = 6). The overall Δ^88/86^Sr_bone–tooth_ difference across all feeding categories is 0.11 ± 0.04‰ (2 s.e., *n* = 40), indicating a significant difference in δ^88/86^Sr between bone and tooth samples (electronic supplementary material, table S4). For ^87^Sr/^86^Sr, there is no trend in the absolute difference in ^87^Sr/^86^Sr between bone and tooth samples (electronic supplementary material, figure S2).

### (b) Calcium isotopes

In total, 118 samples of herbivores, omnivores, insectivores, ovivores, durophages, carnivorous *Varanus* species and carnivorous Crocodilian species were measured (figure 1*c* and electronic supplementary material, table S1). Insectivores have the most positive δ^44/42^Ca = 0.04 ± 0.09‰ (2 s.e., *n* = 8), whereas herbivores yield a significantly (*p* < 0.05) lower mean of −0.11 ± 0.05‰ (2 s.e., *n* = 44). The omnivorous species display significantly (*p* < 0.01) more negative values than both previously mentioned diet groups with a mean of −0.27 ± 0.08‰ (2 s.e., *n* = 18). The ovivorous *Heloderma* specimens display a mean value of −0.19 ± 0.09‰ (2 s.e., *n* = 8), differing significantly (*p* < 0.01) from the insectivorous species. Faunivorous durophages specimens with a mean δ^44/42^Ca of −0.48 ± 0.05‰ (2 s.e., *n* = 13) lie close to carnivorous varanids with an average δ^44/42^Ca of −0.54 ± 0.10‰ (2 s.e., *n* = 13) but differ significantly from all previously mentioned diet groups (*p* < 0.001). The Crocodylia show even more negative values of −0.90 ± 0.13‰ (2 s.e., *n* = 14). Thus, the two carnivorous diet groups of lepidosaurs and archosaurs differ significantly (*p* < 0.001). Overall, the two carnivorous groups yield the most negative δ^44/42^Ca values.

The significant differences between herbivorous and carnivorous feeding categories yield an average trophic-level offset between those groups of −0.44 ± 0.15‰ (herbivores versus varanids) and −0.79 ± 0.17‰ (herbivores versus Crocodylia), respectively. In addition, the insectivorous species are significantly (*p* < 0.05) more positive than the herbivorous species by 0.15 ± 0.13‰ in δ^44/42^Ca.

### (c) Strontium isotopes

Similarly to δ^44/42^Ca, the insectivorous species exhibit the most positive δ^88/86^Sr values (figure 1*d*) with an average of 0.01 ± 0.12‰ (2 s.e., *n* = 7). Herbivorous species show a mean of −0.19 ± 0.05‰ (2 s.e., *n* = 44), which is significantly different (*p* < 0.05) from the insectivorous species. Omnivores (−0.27 ± 0.13, 2 s.e., *n* = 17) and ovivores (−0.20 ± 0.09, 2 s.e., *n* = 8) do not differ significantly from herbivores or each other. Durophagous species show the most negative δ^88/86^Sr values, averaging −0.50 ± 0.05‰ (2 s.e., *n* = 13). In contrast to δ^44/42^Ca, the two carnivorous groups of Varanidae and Crocodylia do not differ in δ^88/86^Sr, with varanids showing −0.37 ± 0.09‰ (2 s.e., *n* = 13) and Crocodylia showing −0.37 ± 0.10‰ (2 s.e., *n* = 14). However, a trophic spacing in δ^88/86^Sr is evident between herbivorous and carnivorous species. The offset is −0.19 ± 0.14‰ between herbivores and varanids (*p* < 0.001) and −0.18 ± 0.15‰ between herbivores and Crocodylia (*p* < 0.01). The offset between insectivores and herbivores is 0.20 ± 0.17‰, with insectivores having significantly more positive values (*p* < 0.05), similar to the pattern observed in δ^44/42^Ca. The radiogenic ^87^Sr/^86^Sr ratio does not show any significant dependence with diet category (electronic supplementary material, figure S3).

## 4. Discussion

### (a) Difference between bone and teeth in δ^44/42^Ca and δ^88/86^Sr

The potential offset in δ^44/42^Ca between bone and tooth remains a topic of debate. Such an offset (in the same individual) can either occur if the isotopic composition of the ingested diet changes significantly between the time of bone and tooth formation, or by distinct physiological isotope fractionation effects during bone or tooth formation. Previous studies reported a systematic offset in δ^44/42^Ca between bone and tooth enamel in mammals, related to the influence of a suckling signal in the enamel calcium, shifting the enamel to more negative δ^44/42^Ca values due to the consumption of isotopically light mother’s milk [44,61,65–67].

However, our dataset does not show an overall difference in δ^44/42^Ca between bone and enamel from the same reptile individual (figure 1*a*), neither for the whole dataset nor for the wild specimens alone (electronic supplementary material, figure S5a). The different results for reptiles compared with mammals might also be explained by further physiological processes such as differences in calcium metabolism, bone remodelling, metabolic rate and tooth replacement patterns (polyphyodont versus diphyodont). Moreover, potential dietary shifts during ontogeny should not be too prominently reflected in an isotopic offset between bone and tooth tissues, except in cases of strong seasonal diet shifts (see electronic supplementary material for details). This is because most of the reptilian species analysed in this study continuously replace their teeth within a few months (e.g. every 10 weeks for *Iguana iguana*, and within a few months for crocodiles [29,30]). Hence, if during adulthood the average diet (reflected in the bone) does not change for prolonged periods between isotopically distinct feeding categories such as faunivory and herbivory or insectivory and carnivory, the teeth should record similar isotope compositions as the bones. Nevertheless, the regular tooth replacement opens the possibility to monitor seasonal dietary changes in reptilian species using calcium isotopes. Although there is some scatter in the dataset, no clear trend is observed. Therefore, we conclude that there is no systematic calcium isotope offset between dental and osseous hard tissues in reptiles.

Nevertheless, reptiles from one feeding category showed a pronounced bone–tooth offset in δ^44/42^Ca. Both specimens of the ovivorous *Heloderma* showed a large offset in Δ^44/42^Ca_bone–tooth_ of −0.31 ± 0.02‰. Ovivores typically crack open calcareous-shelled eggs without ingesting the carbonate eggshell, consuming mostly egg yolk and white [68]. Egg white, in particular, has very positive calcium isotope values (δ^44/42^Ca_NIST915a_ = 1.37 ± 0.01‰) [42]. The δ^44/42^Ca-enriched tooth samples are in line with egg consumption. However, the bone samples display low δ^44/42^Ca values like those of carnivorous varanid species, in agreement with a faunivorous diet [68]. Bone, which is formed and remodelled over the entire lifespan, records a long-term average diet composition, including earlier life stages. In contrast, the permanently replaced teeth may reflect short-term ontogenetic and/or seasonal diet shifts during adulthood, recording dietary preferences shortly before death. Thus, juvenile and growing subadult animals might not have predominantly ingested eggs but rather a carnivorous diet. However, during later life stages, the analysed *Heloderma* individuals were probably feeding on eggs during tooth formation.

Only a few studies deal with stable δ^88/86^Sr isotope values as a dietary proxy for mammals [41,44,55]. Lewis *et al*. [55] focused on enamel samples, whereas Knudson *et al*. [41] analysed bone and enamel samples from the same individuals. Their data showed an average offset in δ^88/86^Sr between bone and teeth of 0.07 ± 0.07‰ (2 s.e., *n* = 35). Guiserix *et al*. [44] found significantly higher δ^88/86^Sr values in bone than in enamel by 0.08‰, although not from the same individuals. They attributed this offset to a suckling effect, similar to calcium isotopes. In contrast, our dataset of extant reptiles cannot be influenced by any suckling effects, but still implies that there is an offset between the δ^88/86^Sr values of bone and teeth from the same individual (figure 1*b*). Covering the whole dataset, bone samples showed on average slightly more positive δ^88/86^Sr values than teeth by 0.11 ± 0.04‰ (2 s.e., *n* = 40), which is within uncertainty in comparison to that offset detected for archaeological human remains [41] and other mammalian species [44]. For the marine feeding algaevorous *A. cristatus* the Δ^88/86^Sr_bone–tooth_ offset is much larger with 0.25 ± 0.07‰ (2 s.e., *n* = 6). This offset points to a shift in δ^88/86^Sr of the diet, however, no such difference occurs in δ^44/42^Ca of the same samples. Furthermore, in contrast to calcium isotopes, no Δ^88/86^Sr_bone–tooth_ offset between bone and tooth is observed for the ovivores (figure 1*b*).

### (b) Stable calcium and strontium isotopes as trophic-level proxies in reptiles

Distinct feeding categories display significant and systematic δ^44/42^Ca differences between herbivores and different faunivorous groups (figure 1*c*). Both carnivorous groups, crocodiles and varanids, have significantly lower δ^44/42^Ca compared with herbivores, consistent with the observed trophic-level effect in calcium isotopes (~0.30‰) for extant mammals and extinct dinosaurs [25,48,51,52]. However, the δ^44/42^Ca offset between herbivore species and the carnivorous varanids (−0.44 ± 0.15‰) is larger than the average trophic-level effect reported in the literature. Varanids, known for consuming a broad range of animal components such as insects, snails, crustaceans and eggs, including small reptiles or mammals in larger species’ diets [69], exhibit less negative δ^44/42^Ca values than Crocodylia, which show an even larger carnivore–herbivore offset (−0.79 ± 0.17‰). Most crocodiles in our study (electronic supplementary material, table S1 and figure S6) are large, generalistic, semi-aquatic carnivores, consuming whole aquatic and/or terrestrial vertebrates, and often being apex predators in their ecosystems. The substantial consumption of bone (rich in calcium and with low δ^44/42^Ca compared with soft tissues [48]) and predation on vertebrates from a higher position in the trophic chain [70] (having lower tissue δ^44/42^Ca values) might explain the significant difference between these carnivorous reptiles. Remarkably, the δ^44/42^Ca offset between varanids and Crocodylia (−0.35 ± 0.22‰) is nearly as large as that between herbivores and varanids and close to the trophic-level effect reported in the literature (~0.30‰), suggesting that Crocodylia occupy an even higher trophic position relative to the varanids included in this study. This is consistent with their role as apex predators which also may prey on other carnivorous animals, e.g. from the aquatic environment with more complex food webs.

However, significant differences in trophic-level are observed between herbivorous, carnivorous and insectivorous species, which exhibit the highest δ^44/42^Ca values of all other feeding categories (figure 1*c*). This corroborates the results of a limited number of insectivorous mammals, showing the most positive δ^44/42^Ca values for insect feeders [50]. The possibility to distinguish insectivorous taxa based on calcium isotopes suggests that this proxy can potentially elucidate dietary transitions not only from carnivory to herbivory but also from insectivory to herbivory, both within an individual’s lifespan and over evolutionary timescales in the fossil record. This has important implications given that herbivory has independently evolved multiple times from faunivory in various vertebrate clades [1,7,8,14].

Omnivorous species exhibit intermediate δ^44/42^Ca values between herbivores and carnivores. However, the substantial ingestion of bioavailable geogenic calcium (e.g. carbonate) from soil attached to soil-dwelling insects (mainly termites) for *Physignathus cocincinus* [71] and *Pogona vitticeps* [72] seems to shift the δ^44/42^Ca (and δ^88/86^Sr) values towards more positive values (electronic supplementary material, figure S6), as expected for insect-feeding species (figure 1*c,d*). Ovivores also show relatively high values, which can be attributed to the consumption of egg yolk and particularly egg white, which is known for its elevated δ^44/42^Ca values [42]. However, based on δ^44/42^Ca alone, a differentiation between ovivores and herbivores is not possible, whereas ovivores significantly (*p* < 0.05) differ from insectivores.

δ^88/86^Sr shows a systematic trophic-level effect, with an offset of −0.19 ± 0.14‰ between herbivorous and carnivorous varanids, smaller than the δ^44/42^Ca offset but in the same direction. The offset between herbivores and the carnivorous Crocodylia is similar at −0.18 ± 0.15‰. Our results indicate that, for reptiles, the trophic-level effect in δ^88/86^Sr is smaller than in δ^44/42^Ca, which is consistent with the limited number of studies using δ^88/86^Sr as a trophic-level proxy in other taxa. Lewis *et al*. [55] reported a Δ^88/86^Sr_diet–teeth_ offset of −0.322 ± 0.060 ‰ (2 s.d.) in a controlled feeding experiment with domestic pigs. Our results align with those for extant African mammals (approximately −0.18‰ [43]) and Late Pleistocene mammalian fossils (approximately −0.14‰, only enamel data from [44]). δ^88/86^Sr values for insectivores show the most positive values (0.01 ± 0.12‰) and are significantly higher (*p* < 0.05) than those of the herbivores (−0.19 ± 0.05‰). This suggests that stable strontium isotopes, like calcium isotopes, can differentiate between herbivorous and insectivorous feeding behaviours. However, due to the much better preservation potential in fossil skeletal remains, calcium isotopes are preferred for dietary reconstruction on fossil specimens over strontium, which is more prone to post-mortem alteration during diagenesis [44–46,48,73].

**Figure 2.**
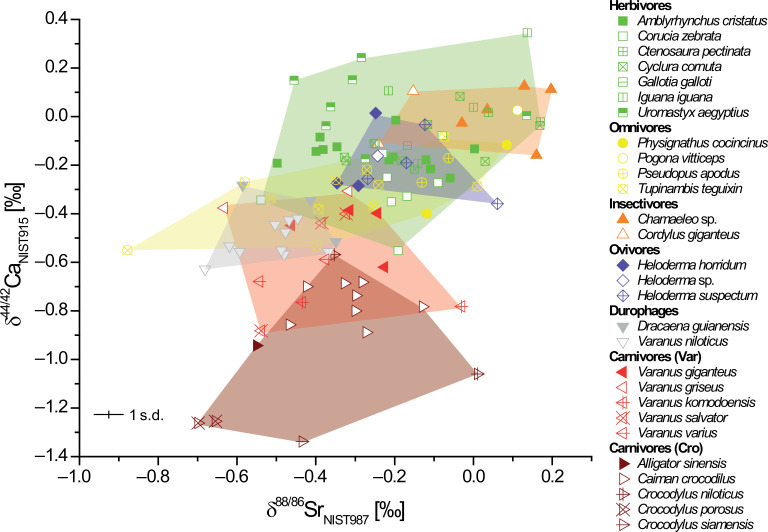
Cross-plot between δ^88/86^Sr and δ^44/42^Ca of modern reptile bone and teeth samples to characterize the isotopic differences between distinct diet groups (electronic supplementary material, table S1) and the trophic spacing between herbivores and faunivores. Note that for carnivores two groups are separately plotted: lepidosaurs (i.e. Varanidae = Var) and archosaurs (i.e. crocodiles = Cro). Mean uncertainties for both isotope systems are provided at the 1 s.d. level.

**Figure 3.**
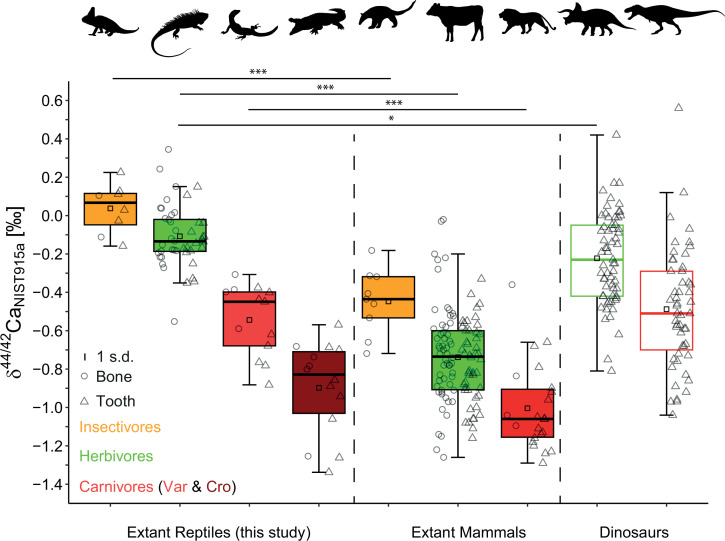
δ^44/42^Ca values for extant reptiles from this study for different feeding categories in comparison to literature data from modern mammals (filled box plots) [42,50,51,53,61,66,74] and dinosaurs (open box plots) [47,48,52]. For dinosaurs, only tooth enamel data was used to minimize the potential effects of diagenesis. Note the highly significant differences in δ^44/42^Ca between reptiles and mammals from the same feeding categories but a similar trophic-level difference. Significance levels are defined as follows: *p < 0.05 ; **p < 0.01; ***p < 0.001. Black silhouettes originate from http://www.phylopic.org.

However, the combination of stable strontium and calcium isotopes as trophic-level proxies can potentially enable refined dietary and trophic-level reconstructions (figure 2). The biplot of δ^44/42^Ca versus δ^88/86^Sr of the same specimens shows a systematic trophic-level effect. Carnivorous species exhibit lower values compared with herbivorous species in both isotope systems, whereas omnivores plot in between, either closer to the herbivore- or carnivore-feeding trait end member, depending on their diet composition. Insectivores consistently show more positive isotope values than all other feeding categories. Considering each isotope system individually (figure 1*c,d* and figure 3) as well as in combination (figure 2), calcium isotopes appear to be a more effective dietary proxy for assessing the trophic position of an animal. Calcium isotopes seem to better characterize feeding differences among modern-day reptiles, allowing for more precise identification of apex predators among carnivores and distinguishing insectivores from other feeding traits. Nevertheless, the δ^88/86^Sr has the potential to further refine dietary reconstructions due to slightly different fractionation behaviour for certain feeding categories. Applying this toolbox to fossils, diet-related calcium isotope compositions are more resistant to post-mortem alteration by diagenesis, particularly in enamel [24,45,46,48], compared with strontium isotopes [44].

There are only two studies [43,44] combining these two isotope systems so far. Whereas Guiserix *et al*. [44] used both calcium and strontium isotopes, as well as zinc isotopes, for an Upper Pleistocene fossil assemblage, Tütken *et al*. [43] studied extant African mammals. Diagenetic alteration might have affected the relationship between strontium and calcium isotopes in the fossil specimens, however, the modern mammalian specimens were not affected by diagenesis, as shown by the reptilian dataset presented in this study. The direct comparison of both extant datasets for herbivores, carnivores and insectivores (electronic supplementary material, figure S8) shows that both yield a significant positive linear relationship between calcium and stable strontium isotopes, while the slope of the regression is steeper for the mammalian data. The different slopes might indicate differences in the trophic-level effect between reptilian and mammalian carnivores.

### (c) ^87^Sr/^86^Sr as a proxy for bioavailability of ingesta

Besides the potential to reconstruct the provenance and mobility of vertebrates during foraging (see electronic supplementary material for details), the analysis of ^87^Sr/^86^Sr also allows identifying species which are feeding on marine resources. The algaevorous *Amblyrhynchus cristatus* shows ^87^Sr/^86^Sr values consistent with modern-day seawater ^87^Sr/^86^Sr of 0.70918 [64] in both bones and teeth (electronic supplementary material, figure S7). This species is the only lepidosaur feeding on marine resources [75], scraping algae from basaltic rocks (with a mantle-derived ^87^Sr/^86^Sr of ~0.703 in nearshore waters of the Galapagos Islands [76]). During food consumption, not only algae but also silicate particles from the basaltic rocks are ingested, as indicated by rough, durophage-like dental microwear textures [10]. However, despite the intake of rock material, the silicate-bound strontium was not bioavailable, as otherwise the ^87^Sr/^86^Sr would be shifted away from the seawater value to lower ^87^Sr/^86^Sr ratios of the mantle-derived strontium from the basalt, which is not the case (electronic supplementary material, figure S7).

### (d) Comparison between stable calcium and strontium isotope data and DMTA

DMTA is a method to quantify (microscopic) wear patterns on dental enamel with specific surface roughness parameters for diet reconstruction. The wear patterns are related to ingesta abrasivity, reflecting material properties of the diet consumed, but likely also diverse other aspects such as bite force, tooth shape, mastication mode and more [77,78]. As DMTA is independent of the diet’s chemical composition, it can infer the physical properties of the diet rather than the trophic position of the consumer. However, several DMT parameters that consider overall surface roughness (i.e. height and volume parameters) cannot distinguish between carnivory and herbivory, and between omnivory, insectivory and herbivory in extant lepidosaurs [10].

**Figure 4.**
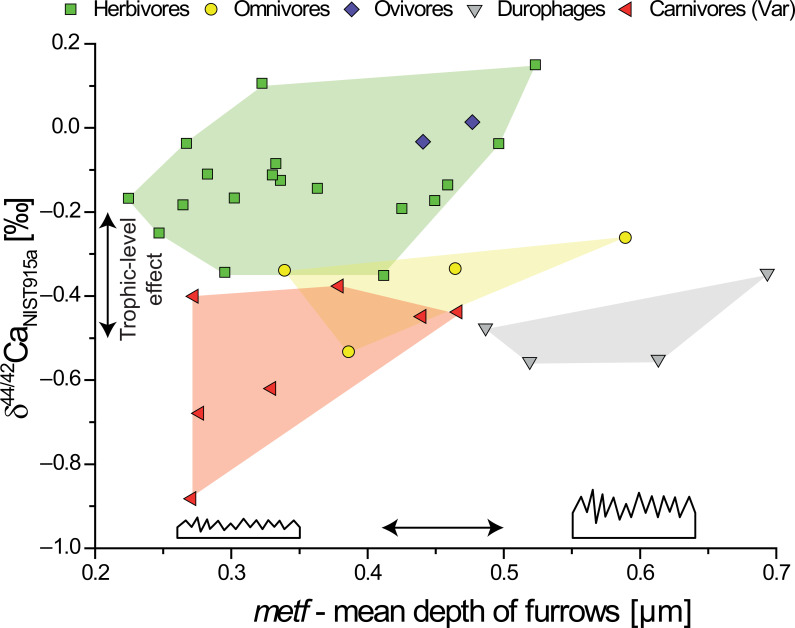
Dental microwear texture parameter mean depth of furrows (*metf*) of extant lepidosaurs from Winkler *et al*. [10] plotted against tooth δ^44/42^Ca of the same individual. Analytical uncertainty for δ^44/42^Ca (0.03, 1 s.d.) is smaller than the individual data points. Note that *metf* enables to differentiate durophages faunivores from carnivores with the former having deeper furrows but similarly low δ^44/42^Ca as the latter.

By using multiple dimensions of DMT parameters (principle component analysis (PCA), biplots) a better resolution between general trophic categories can be achieved. Specifically, the combination of mean density (*medf*) versus mean depth (*metf*) of furrows on the enamel surface can separate carnivorous from herbivorous/omnivorous/insectivorous lepidosaurs and distinguish these groups from durophagous lepidosaurs [10]. Thus, combining these DMT parameters with chemical proxies that relate to trophic-level differences bear the potential to further refine dietary reconstructions. Here, we combine these mechanical and chemical dietary proxies on the same individuals for the first time (figure 4 and electronic supplementary material, figures S10, S11 and S13). Using δ^44/42^Ca, both carnivorous lepidosaurs and crocodilians can be distinguished from herbivorous lepidosaurs (figure 1*a*–d). However, specialized faunivorous taxa (ovivores and durophages) fell either within the calcium isotope range (figure 2) and/or DMT parameter range [10] of herbivores or carnivores, respectively. However, by combining δ^44/42^Ca and DMT parameter *metf* (figure 4), faunivorous durophages could be distinguished from both carnivores and herbivores. Similarly, the combination of δ^44/42^Ca and *medf* (electronic supplementary material, figure S10*b*) can separate ovivores from carnivores. However, even with this combined approach, it is not possible to clearly separate omnivores from herbivores or carnivores. While not as clear as for δ^44/42^Ca, the combination of DMTA and δ^88/86^Sr data (electronic supplementary material, figure S11) is able to distinguish between ovivores and durophages as well, but does not add further information in comparison to δ^44/42^Ca. However, the PCA including DMTA and stable isotope data (electronic supplementary material, figure S13) shows that δ^88/86^Sr is negatively correlated with the majority of DMT parameters, spanning PC1.

Overall, the application of both mechanical (DMTA) and chemical (δ^44/42^Ca) dietary proxies in a multi-proxy approach offers the potential for a more accurate and refined reconstruction of dietary preferences and feeding habits in extant reptiles. This toolbox can potentially be applied to other vertebrate clades with durophages [17,79] for more refined dietary reconstructions, as well as for extant [78] and extinct reptilian taxa such as pterosaurs [80] or dinosaurs [20,21], for which so far only DMTA has been analysed, for instance, to assess the degree of bone consumption among theropods [21]. In the future, this toolbox can be further expanded by adding other diet-related isotope systems with deep time preservation potential, such as enamel-bound nitrogen [33] or zinc [44,81] isotopes, to even better resolve the types and amounts of food resources used and to reconstruct the trophic ecology and niche partitioning of fossil reptiles and other vertebrates.

### (e) Implications for dietary reconstructions of fossil reptiles

For studies aiming to reconstruct palaeo-diet and palaeo-food webs, a framework of modern reference data and calibrations of dietary proxies are essential. Although non-traditional isotopes are increasingly used as dietary proxies for modern and fossil mammals [25,44,55,67], few studies focus on diet reconstructions of extinct reptiles, such as dinosaurs [51,52]. Furthermore, a robust reference frame of stable calcium and strontium isotope data for modern reptiles has been lacking.

This study shows that the observed trophic-level effect between diet and hard tissues of modern mammals [42,51] is of similar magnitude in extant reptiles, allowing its application to fossil reptilian taxa. However, the absolute δ^44/42^Ca values for each diet group significantly differ between extant reptilian and mammalian species (figure 3). Both modern datasets are compilations across a variety of ecosystems (electronic supplementary material, figure S1). Therefore, strong local baseline effects for a single ecosystem can be excluded and differences in physiology (e.g. viviparous versus oviparous or calcium metabolism) should be considered as the major controlling factor of the observed isotope offset. However, comparing mammalian and reptilian δ^44/42^Ca data with dinosaur data, it becomes obvious that the dinosaur δ^44/42^Ca data of different diet categories are closer to the values of modern reptiles rather than mammals (figure 3). The limited number of δ^44/42^Ca data of extant birds (ostrich bones, δ^44/42^Ca = −0.21 ± 0.02 ‰, 2 s.e., *n* = 6 [48]; chicken bones, δ^44/42^Ca = −0.58 ± 0.08 ‰, 2 s.d., *n* = 1 [42] and δ^44/42^Ca = −0.13 ± 0.07 ‰, 2 s.d., *n* = 1 [53]) shows a general agreement with the results obtained from herbivorous reptiles and dinosaurs. Notably, insectivorous reptiles showed significantly more positive δ^44/42^Ca and δ^88/86^Sr values than herbivores and carnivores (figure 2). This provides an opportunity to determine transitions from insect- to plant-feeding during both ontogeny and evolution, facilitating the assessment of high-fibre herbivory evolution from the earliest insectivorous reptiles and non-mammalian synapsids [7,9].

Our study also indicates no systematic δ^44/42^Ca offset between bone and enamel samples from the same individual (except for ovivorous *Heloderma* spp.). Bones, which record long-term diet-related δ^44/42^Ca, are more frequently and easily available for minimal-invasive sampling compared with tooth enamel, although only sub-milligram amounts of both phosphatic hard tissues are required for calcium isotope analysis. The systematic calcium isotope fractionation along the food chain enables us to identify carnivores, including apex predators, and distinguish them from herbivores. Furthermore, original diet-related calcium isotopes have long-term preservation potential due to the high concentration of calcium in hydroxylapatite, even in fossil bones [24,46,48], enabling dietary reconstruction of extinct taxa and fossil food webs. In contrast, strontium, as a trace element, is more prone to post-mortem alteration [46,54,82]. Post-mortem diagenetic uptake of strontium with geogenic positive δ^88/86^Sr values [83] will cause the loss of diet-related trophic fractionated negative δ^88/86^Sr. This limits the application of stable strontium isotopes as dietary proxy in fossil material [44,73], but makes δ^88/86^Sr a sensitive proxy for the identification of diagenetically altered fossil specimens (figure 4).

## 5. Conclusion

Diapsid reptiles show a systematic dependence between their hard tissue δ^44/42^Ca and δ^88/86^Sr values and their diet. Both stable isotope systems systematically decrease along the food chain (i.e. lower δ^44/42^Ca and δ^88/86^Sr) and thus enable to efficiently differentiate between diet categories and identify trophic-level differences. Insectivores show the highest values for both calcium and stable strontium isotopes, significantly distinguishing them from other diet categories such as herbivores and faunivores (carnivores, ovivores and durophages). The trophic-level difference in δ^44/42^Ca between herbivores and carnivores is similar to previous studies of mammalian taxa. In contrast, the absolute δ^44/42^Ca values of reptilian species per dietary category are more positive than for their respective counterpart from extant mammalian species. In addition, carnivorous crocodiles show an even larger offset in δ^44/42^Ca from herbivores than carnivorous varanids, reflecting their higher trophic position as apex predators.

No systematic offset in δ^44/42^Ca was detected between bone and tooth samples from the same individuals, for almost all dietary groups, except for the ovivorous *Heloderma*, where teeth had about 0.3‰ higher values than bone samples, likely indicating an ontogenetic dietary shift. Thus, δ^44/42^Ca isotopes of both, bones and teeth, seem to similarly record the diet of reptiles. For δ^88/86^Sr, a slight offset between bone and tooth samples was detected, with bone being more positive by 0.11 ± 0.04‰.

Furthermore, this study demonstrates that combining stable isotope analysis with DMTA of the same individuals can refine our understanding of feeding traits across species. This combination allows for the identification of hard-object feeders (i.e. durophages) and distinguishes them from other dietary categories. This allows characterizing dental wear differences within a single trophic category, thereby further refining dietary reconstructions. Additionally, the analysis of ^87^Sr/^86^Sr of bone–tooth pairs can potentially help constrain (seasonal) habitat use and migration patterns of terrestrial reptiles.

Ultimately, stable calcium and strontium isotope data from modern reptiles of distinct feeding categories, combined with previously published dental microwear textures of the same specimens, provide a modern-day reference frame for dietary reconstructions of extinct reptiles. This multi-proxy toolbox, which can be expanded with further diet-related isotope systems, opens new research avenues to investigate the evolution of herbivory in early synapsids during the Permo-Carboniferous and reconstruct fossil food webs and niche partitioning of extinct non-mammalian species such as dinosaurs and synapsids.

## Data Availability

The dataset supporting this article have been uploaded as part of the electronic supplementary material [84].
